# Placental hypoplasia and vascular malperfusion are associated with reduced brain volumes in fetal congenital heart disease

**DOI:** 10.1038/s41390-025-04698-3

**Published:** 2026-01-07

**Authors:** Claire Baldauf, Valeria Rosas, Paul Zamiara, William T. Reynolds, Shuo Wang, Nhu Tran, Rafael Ceschin, Melissa L. Wilson, Jodie K. Votava-Smith, Jessica L. Wisnowski, Vidya Rajagopalan

**Affiliations:** 1 Fetal and Neonatal Institute, Division of Neonatology, Department of Pediatrics, Children’s Hospital of Los Angeles, Keck School of Medicine, University of Southern California, Los Angeles, CA USA; 2 Division of Cardiology, Department of Pediatrics, Children’s Hospital of Los Angeles, Keck School of Medicine, University of Southern California, Los Angeles, CA USA; 3 Department of Pathology, Children’s Hospital of Los Angeles, Keck School of Medicine, University of Southern California, Los Angeles, CA USA; 4 Department of Biomedical Informatics, University of Pittsburgh, Pittsburgh, PA USA; 5 Department of Radiology, University of Pittsburgh, Pittsburgh, PA USA; 6 Division of Disease Prevention, Policy, & Global Health, Department of Population & Public Health Sciences, Keck School of Medicine, University of Southern California, Los Angeles, CA USA

## Abstract

**Background:**

Critical congenital heart disease (CHD) is associated with poor neurodevelopmental outcomes and long-term psychosocial morbidity. The pre-natal environment is increasingly recognized as a critical effector. However, the association of specific placental lesions with fetal brain volume in CHD remains unexamined.

**Methods:**

This was a multi-site prospective, nested case-control study, that recruited pregnant women with typical fetal development and prenatally diagnosed fetal CHD. Fetal brain MRI was performed at 32.8 ± 3.0 weeks gestation. Placentas were examined pathologically after delivery. Clinical characteristics were gathered from self-reports and medical charts. Multiple linear regression modeling was performed in R with *p* < 0.05 considered significant.

**Results:**

Placental hypoplasia (weight <10th %ile) was associated with lower total fetal intracranial volume in CHD (*p* = 0.006) and combined (CHD + non-CHD) cohorts (*p* = 0.001). Maternal and/or fetal vascular malperfusion was also associated with lower total fetal intracranial volume in combined (*p* = 0.026), and non-CHD cohorts (*p* = 0.020). Presence of any placental pathology was associated with reduced total fetal intracranial volume (*p* = 0.047), an effect moderated by the presence of CHD (interaction term *p* = 0.037).

**Conclusion:**

Placental hypoplasia, vascular malperfusion, and the presence of any placental pathology are associated with decreased total intracranial volumes in-utero. Fetuses with CHD and concomitant placental lesions appear particularly susceptible.

**Impact:**

- Placental vascular malperfusion is associated with reduced fetal intracranial volume.
- Placental hypoplasia (weight <10th %ile) is associated with smaller intracranial volumes in fetal congenital heart disease.
- Placental pathologies uniquely affect in-utero brain development in congenital heart disease.
- This study is the first to link fetal brain development to specific placental pathologies using standardized, reproducible criteria (Amsterdam) with comparative inclusion of a non-congenital heart disease cohort.
- Evidence builds that the in-utero environment impacts neurodevelopment in congenital heart disease; this study points to specific placental lesions that may mediate the pathophysiology underlying these observations.

## Introduction

Congenital heart disease (CHD) affects nearly 40,000 infants per year in the United States^1^ with approximately 25% requiring surgical intervention in infancy.^2^ Poor neurodevelopmental outcomes in these children are common,^3,4^ in addition to long-term psychosocial issues such as anxiety, depression, and reduced educational and economic attainment.^5,6^ Previously thought to be secondary to insults such as cardiopulmonary bypass and prolonged hospitalization, the influence of post-natal factors only accounts for approximately one third of the observed variability in mortality and poor neurodevelopment.^7,8^ The in-utero environment is increasingly implicated, with factors such as low birth weight and prematurity being strong independent risk factors for poor long-term outcomes.^9–12^. Furthermore, early post-natal brain imaging in these infants shows that brain abnormalities in CHD are present prior to surgery and prolonged hospitalization.^13–15^

A critical mediator of the in-utero environment, the placenta has been found to have a high incidence of histopathological abnormalities in CHD pregnancies. These include maternal and fetal vascular malperfusion, infarction, abnormal/dysmature villi,^16–18^, and small for gestation age placental weight.^19^ The severity of histopathologic placental lesions among neonates with CHD has been associated with brain injury and volumetric changes in early post-natal, presurgical brain MRIs.^20^ In-vivo functional imaging studies of the CHD placenta via blood oxygen level dependent MRI demonstrate decreased oxygenation and abnormal intrinsic placental function in these pregnancies.^21–24^ Despite evidence that the in-utero environment is a vital determinant of neurodevelopment in critical CHD, the association of discrete placental diagnoses with fetal intracranial volumes, a strong predictor of subsequent neurodevelopment,^25–27^ remains unexamined. Prior studies investigating placental histopathologic associations with the CHD brain have limitations, including scoring systems adapted from the hypoxic ischemic encephalopathy literature,^20,28^ and lack of a control group.^29^ Thus, we do not know which CHD-specific patterns of clinically reported placental lesions have the most impact on early brain development. Poor fetal brain growth in CHD and other diseases, is a well-documented precursor to later adverse neurodevelopment such as cerebral palsy, and reduced cognitive and intelligence quotient scores, even at 9–10 years of age.^30,31^

In this study, we investigated whether specific placental pathologies impact the fetal brain differently in CHD compared to non-CHD. We aimed to fill current gaps in the literature by examining an earlier imaging timepoint during the pre-natal period, utilizing the Amsterdam criteria, a standardized clinical placental pathology scoring system^32^, and using a prospectively enrolled control group (henceforth referred to as “non-CHD”). Our primary outcome was total fetal intracranial volume, with a secondary outcome of regional fetal brain volumes. Prevalence of maternal risk factors (such as diabetes, obesity, hypertension, etc.) in pregnancy is increasing in the United States^33^ and abroad,^34–36^ with even higher prevalence rates in pregnancies complicated by fetal CHD.^37^ Therefore, we collected and controlled for these factors.

## Methods

This was a prospective, nested, case-control study conducted between 2016 and 2024 at two large cardiac centers: Children’s Hospital Los Angeles (CHLA) and the University of Pittsburgh Medical Center (UPMC). The parent study was approved by the institutional review board at each center (CHLA IRB#: CHLA-22-00324 effective approval 07/2023, CHLA-17-00292 effective approval 06/2024, UPMC Study20030213 effective approval 04/2020) and all subjects signed a written informed consent for participation.

### Study subjects

At Children’s Hospital Los Angeles (CHLA), pregnant women carrying a fetus with prenatally diagnosed congenital heart disease (CHD) were recruited from the CHLA fetal maternal center, a high-risk obstetrics outpatient clinic, or the CHLA cardiology clinic, where they received fetal echocardiograms. Women carrying non-CHD fetuses were recruited via referral from community partner outpatient clinics as well as flyers and social media advertisements. All women were recruited between 21- and 38 gestational weeks.

At UPMC, recruitment for the CHD group took place at one of three clinics: The high-risk pregnancy center at Magee Women’s Hospital, fetal cardiology clinic at Children’s Hospital of Pittsburgh, or an outside affiliate cardiology clinic in Wexford, Pennsylvania. For the non-CHD group, recruitment utilized flyers, a research participation database from the University of Pittsburgh, or were women that received a fetal echocardiogram due to elevated fetal CHD risk (family history) and who had normal results.

**Fig. 1:**
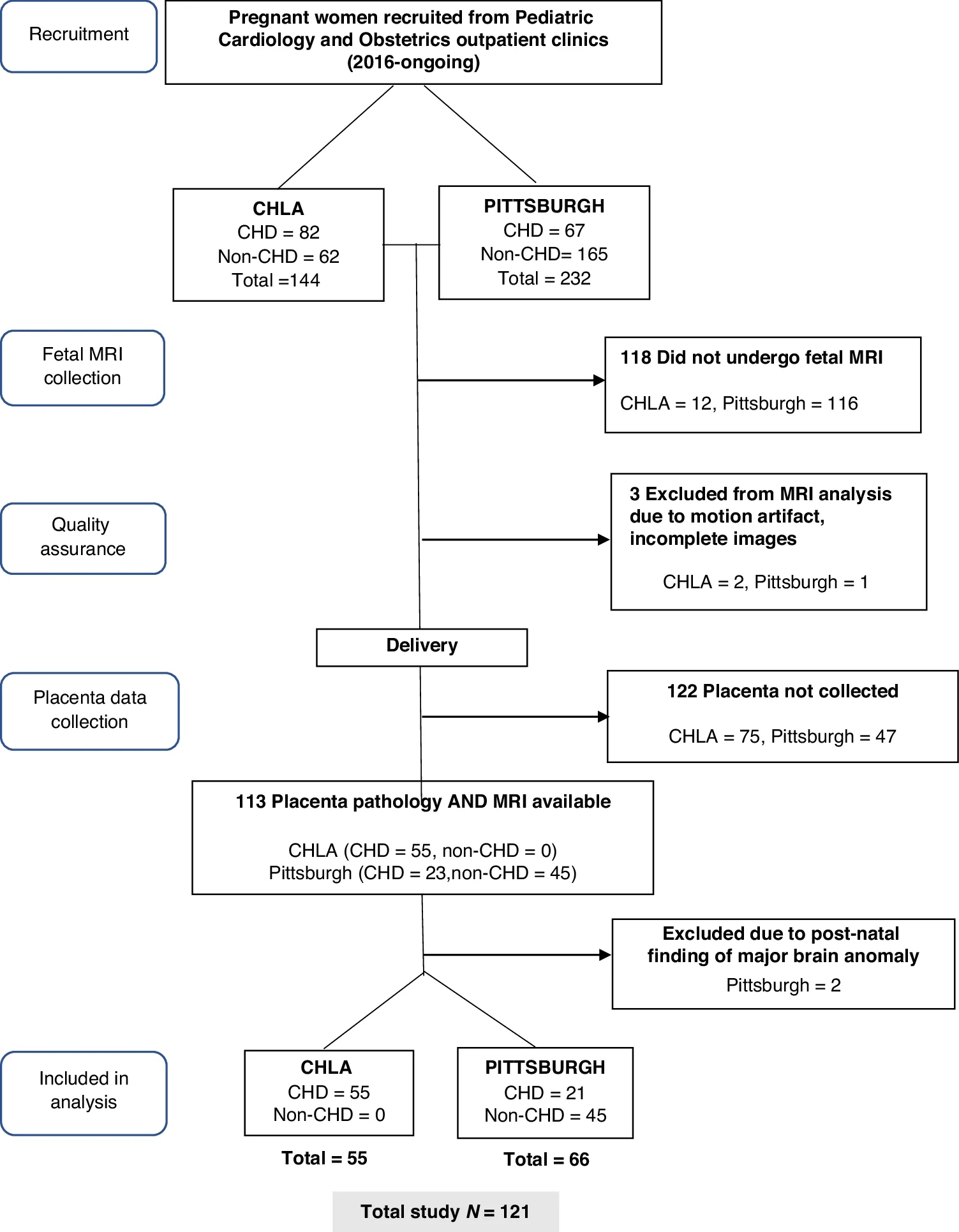
Study STROBE diagram. CHLA Children’s Hospital Los Angeles, CHD Congenital Heart Disease.

Inclusion criteria for the CHD group were a diagnosis on fetal echocardiogram of critical CHD, defined as those fetuses needing cardiac interventional procedures during infancy. For the non-CHD group women needed to be pregnant with a fetus without known CHD. Eligibility included maternal age between 18 and 45 years. Exclusion criteria for both groups were a major anatomical brain anomaly on either prenatal ultrasound or fetal brain MRI, or prenatal diagnosis of a severe/lethal condition (for CHD group, one not associated with their CHD diagnosis). Exclusion criteria for the non-CHD group included babies with a known genetic condition. The study strobe diagram is presented in Fig. 1.

### Image Acquisition

Pregnant women underwent MRI between 24- and 38-weeks’ gestation. Women were placed in a supine position. No sedatives or contrast were given. Scanning took place for no more than 60 minutes depending on fetal movement. At CHLA, a 3-Tesla Philips Ingenia scanner (Philips, Bothell, WA) was used. At CHP, a 3-Tesla Siemens Skyra scanner (Siemens, Ehrlangen Germany). Structural T2 imaging (SS-FSE/HASTE) was performed at both sites with the following harmonized parameters: TE = 160 ms, TR = 9000–12,000 ms, 3 mm slice thickness, no interslice gap, 1 × 1 mm in plane resolution. Fetal brains were scanned in each of three planes, three times, resulting in nine images per subject and images were repeated if excessive motion was present.

### Image analysis

Fetal subjects' T2 MR images were reconstructed into volumetric T2 images in a manner consistent with a previous publication by our group.^38^ Reconstructed fetal brains were then processed through a volumetric parcellation tool previously validated in the same publication. Briefly, fetal brains were parcellated into regions including cortical plate, white matter, hippocampus-amygdala complex, deep grey matter, cerebellum, brainstem, extra-axial cerebrospinal fluid, and intraventricular cerebrospinal fluid. Regions were then combined to form a total intracranial volume (ICV). Finally, regional volumes were normalized to ICV as a ratio (i.e., regional volume / ICV).

### Clinical data collection

At Children’s Hospital Los Angeles, at the time of a fetal MRI scan, subjects filled out a questionnaire regarding their pregnancy and medical history. For non-CHD subjects, pre-pregnancy body mass index (BMI), smoking status (either smoking during pregnancy or no), hypertension in pregnancy, and diabetes in pregnancy were obtained via maternal report on the questionnaire. Birth measurements for non-CHD subjects were obtained via follow-up phone call. For CHD subjects, consent included review of the medical chart, both at the delivery hospital and CHLA. Postnatal factors such as birth history information were extracted from either the delivery record (when available) or the CHLA medical record. No discrepancies in self-reported pregnancy complications and chart reviewed records were found. At UPMC, maternal and infant data were collected from the Magee Obstetric Maternal and Infant Database, or from the medical record.

### Cardiac lesion categorization

Cardiac lesion categorization was defined based upon post-natal echocardiography reports by a pediatric cardiologist (JVS). Lesions were subdivided further by ventricle, either 1-ventricle or 2-ventricle physiology. For cohort description purposes, JVS also categorized subjects by specific lesion type (Supplementary Table 1).

### Maternal risk factors in pregnancy

Women diagnosed with pre-eclampsia, HELLP syndrome, chronic hypertension, or gestational hypertension, were considered positive for a hypertensive disorder of pregnancy. Subjects were considered obese if their pre-pregnancy, or first trimester (12 completed gestational weeks or less) body mass index (BMI) was greater than or equal to 30.0. Maternal BMI for the CHLA subjects was collected from the CHLA medical record, during the fetal MRI study visit, or from the delivery hospital medical record. At UPMC, subject BMI was obtained from the clinical record with gestation at time of measurement denoted. Any form of diabetes mellitus in pregnancy, including type 1, type 2, or gestational diabetes, was considered positive for maternal diabetes. For all women included in this study, the presence of maternal diabetes, hypertensive disorder of pregnancy, maternal obesity, and maternal smoking status was analyzed dichotomously as either present or absent.

### Placental pathology

For CHLA subjects, clinical examination of the placenta took place at either CHLA or at Hollywood Presbyterian Medical Center (a delivery hospital) per standard clinical protocols. Placentas examined at CHLA were either transported with the infant or were couriered to CHLA after delivery for pathological exam. At UPMC, placentas were evaluated in house by pathologists using their standard clinical protocol. All placental weights were obtained on fresh, trimmed samples prior to sampling of placental parenchyma.

Pathology reports were reviewed and categorized by CB and independently reviewed and scored by PZ, a placentally trained board-certified pathologist. Reviewers were blinded to cohort (CHD or non-CHD) status. The inter-rater reliability calculated by percent agreement on data points was 94.4%. A consensus agreement through discussion of the clinical report details was reached by PZ and CB for differentially scored placental lesions. The Amsterdam Criteria with implementation guided by Redline et al.^32^ was used.

Placental variables examined were maternal vascular malperfusion, fetal vascular malperfusion, villitis of unknown etiology, avascular villi, chorangiosis, and placental weight percentile.^39^ Additional placental variables created were: ”placental hypoplasia”, defined as a placental weight below the 10th percentile, “placental efficiency,” defined as birth weight divided by placental weight (both in grams), and “any pathology” defined as the presence of any one or more identified placental abnormalities. In line with other published studies^18^ and to maintain statistical power, we chose to create a combined variable encompassing fetal and/or maternal vascular malperfusion, “any vascular malperfusion.” All placental variables were dichotomous except for placental weight percentile and placental efficiency, which were continuous.

### Statistical analyses

All statistical analysis was performed using R software (version 4.3.0, R Core Team, 2023). Demographic and clinical characteristics are given as mean ± standard deviation for continuous variables and median with interquartile range for categorical variables. The primary outcome variable was total intracranial volume. The effect of placental abnormalities on outcome in CHD and non-CHD fetuses was examined using heteroscedasticity robust, linear regression modeling. Gestational age at MRI, site (CHLA or UPMC), and fetal sex are established covariates for fetal brain development and were included in all models. We then iteratively included other known confounders of placental pathology and/or brain volume. Only those that changed the beta coefficient by more than 10% were included in the final model. We tested each individual placental lesion for association with total intracranial volume. This was done for regionalized brain volumes as secondary outcome variables in the same manner. All models were adjusted for the following covariates: cohort (CHD or non-CHD), maternal hypertensive disorder of pregnancy, obesity, and smoking during pregnancy. Stratified examination by cohort status was also performed. Due to significant effects of both placental weight and presence of vascular malperfusion, a post-hoc analysis of the relationship between these two variables was performed and reported. To address the lack of non-CHD subjects from the CHLA site, a sensitivity analysis was performed to look for potential confounding by site. The main effect of change in ICV was re-estimated using only UPMC subjects which allowed us to assess whether associations between placental pathology and fetal ICV persisted when analyses were performed at only one site. Statistical analyses were supervised by a statistician for accuracy (MLW). Alpha was set at 0.05.

## Results

### Cohort characteristics

Patient characteristics by cohort (CHD, *N* = 76 versus non-CHD, *N* = 45) are shown in Table 1. Most of the CHD cohort was from CHLA (72%); all non-CHD subjects were from UPMC. A larger percentage of the non-CHD cohort had a hypertensive disorder in pregnancy versus the CHDs (29% vs 9%), a reflection of difference in site proportion as UPMC subjects had a 24% rate of hypertensive disorders versus 7% at CHLA. Maternal race and ethnicity were different in the two cohorts, also due to differences in site. Fetal sex demonstrated a higher proportion of the CHD cohort were males versus the non-CHDs.

**Table 1:** Patient Characteristics by Cohort

Characteristic	Overall *N* = 121^*a*^	CHD *N* = 76^*a*^	Non-CHD *N* = 45^*a*^	*p*-value^*b*^
**Site**				<0.001
Children’s Hospital L.A.	55/121 (45%)	55/76 (72%)	0/45 (0%)	
University of Pittsburgh	66/121 (55%)	21/76 (28%)	45/45 (100%)	
**Smoker**	7/121 (5.8%)	3/76 (3.9%)	4/45 (8.9%)	0.4
**Hypertensive disorder**	20/121 (17%)	7/76 (9.2%)	13/45 (29%)	0.005
**Diabetes Mellitus**	30/121 (25%)	18/76 (24%)	12/45 (27%)	0.6
**Obesity**	42/121 (35%)	22/76 (29%)	20/45 (44%)	0.084
**Race**				<0.001
Asian or Pacific Islander	10/121 (8.3%)	9/76 (12%)	1/45 (2.2%)	
Black	15/121 (12%)	5/76 (6.6%)	10/45 (22%)	
Other	23/121 (19%)	23/76 (30%)	0/45 (0%)	
White	73/121 (60%)	39/76 (51%)	34/45 (76%)	
**Ethnicity**				<0.001
Hispanic or Latino	26/119 (22%)	26/74 (35%)	0/45 (0%)	
Not Hispanic or Latino	93/119 (78%)	48/74 (65%)	45/45 (100%)	
(Missing)	2	2	0	
**Genetic disorder**	8/121 (6.6%)	8/76 (11%)	0/45 (0%)	0.025
**Gestational age at MRI (weeks)**	32.8 (3.0)	33.3 (3.0)	31.9 (2.8)	0.011
**Sex**				0.039
Female	50/121 (41%)	26/76 (34%)	24/45 (53%)	
Male	71/121 (59%)	50/76 (66%)	21/45 (47%)	
**Gestational age at birth (weeks)**	38.7(1.5)	38.8(0.9)	38.6(2.1)	0.6
**Birth weight (g)**	3,211.4(568.6)	3,226.4(537.1)	3,185.5(624.9)	0.9
(Missing)	1	0	1	
**Birth Length (cm)**	49.9 (2.9)	49.5 (2.8)	50.7 (3.1)	0.015
(Missing)	3	2	1	
**Birth Head circumference (cm)**	33.9 (1.9)	33.9 (1.7)	34.0 (2.1)	0.7
(Missing)	3	2	1	
**C-section delivery**	59/121 (49%)	39/76 (51%)	20/45 (44%)	0.5

^*a*^n/*N* (%); Mean(SD)

^*b*^Pearson’s Chi-squared test; Fisher’s exact test; Wilcoxon rank sum test

Our CHD cohort was comprised of 76 subjects with complex heart defects. 2-ventricle defects made up 47% and 1-ventricle defects 53%. For a list of all cardiac lesions included in our CHD cohort, please see Supplementary Table 1.

### Placental characteristics by cohort

lacental weight and rate of placental hypoplasia were similar between CHD and non-CHD cohorts. Maternal vascular malperfusion was more common in the non-CHD cohort (16% vs 9%), and fetal vascular malperfusion was more common in the CHD cohort (14% vs 8.9%). Avascular villi, a finding related to fetal vascular malperfusion, were more common in the CHD group (21% vs 11%). Placental characteristics by cohort are reported in Table 2.

**Table 2:** Placental Characteristics by Cohort

Characteristic	Overall *N* = 121 ^*a*^	CHD *N* = 76 ^*a*^	Non-CHD *N* = 45 ^*a*^	p-value ^*b*^
**Placental Weight (g)**	455.1(97.9)	451.9(94.2)	460.3(104.6)	0.7
**Placental Weight %ile**	30.0(30.9)	28.9(30.1)	31.9(32.3)	0.7
**Placental Hypoplasia**	50/121 (41%)	31/76 (41%)	19/45 (42%)	0.9
**Placental Efficiency**	7.2 (1.4)	7.3 (1.5)	7.0(1.1)	0.2
**Acute Chorioamnionitis**	25/121 (21%)	12/76 (16%)	13/45 (29%)	0.085
**Maternal Vascular Malperfusion**	14/121 (12%)	7/76 (9.2%)	7/45 (16%)	0.3
**Fetal Vascular Malperfusion**	15/121 (12%)	11/76 (14%)	4/45 (8.9%)	0.4
**Any Vascular Malperfusion**	28/121 (23%)	18/76 (24%)	10/45 (22%)	0.9
**Chorangiosis**	31/121 (26%)	18/76 (24%)	13/45 (29%)	0.5
**Avascular villi**	21/121 (17%)	16/76 (21%)	5/45 (11%)	0.2
**Villitis of Unknown Etiology**	13/121 (11%)	7/76 (9.2%)	6/45 (13%)	0.5
**Any Pathology Present**	76/121 (63%)	46/76 (61%)	30/45 (67%)	0.5

^*a*^Mean(SD); n/N (%)

^*b*^Wilcoxon rank sum test; Pearson’s Chi-squared test; Fisher’s exact test

### Total fetal intracranial volume and placental pathology

Table 3 lists regression model parameters for associations between placental lesions, cohort, and total fetal intracranial volume (ICV) with inclusion of an interaction term between cohort and placental lesion in predicting ICV. Combined cohort (both CHD and non-CHDs) and analyses stratified by cohort are presented below:

**Table 3:** Main effect of placental lesions (independent variable) on total fetal intracranial volume (ICV) (dependent variable, measured in mm^3^)

Placental Lesion	Main Effect on Total Intracranial Volume		Cohort (CHD vs non-CHD)		Interaction	
	Beta	*p*	Beta	*p*	Beta	*p*
**Placental weight percentile**	3.31 × 10^2^	0.041*	−256 × 10^3^	0.780	−0371	0.999
Placental hypoplasia	−172 × 10^4^	0.099	−136 × 10^3^	0.873	−6.02 × 10^3^	0.650
Placental efficiency	8.11 × 10^2^	0.869	1.06 × 10^4^	0.793	−196 ×10^3^	0.727
**Any pathology present**	−229 × 10^4^	0.047*	−228 × 10^4^	0.044*	2.95 ×10^4^	0.037*
Acute chorioamnionitis	7.70 × 10^3^	0.515	−128 ×10^2^	0.987	−152 ×10^4^	0.352
**Any vascular malperfusion**	−288 × 10^4^	0.026*	−963 × 10^3^	0.204	2.74 × 104	0.086
Maternal Vascular Malperfusion	−163 × 10^4^	0.270	−4.58 ×10^3^	0.525	−0.357	0.999
**Fetal vascular malperfusion**	−4.16 × 10^4^	0.028*	−8.57 × 10^3^	0.223	5.07 × 10^4^	0.021*
Chorangiosis	−101 × 10^4^	0.410	−994 × 10^3^	0.211	2.47 × 10^4^	0.115
Avascular villi	−7.61 × 10^3^	0.656	−3.44 × 10^3^	0.640	3.09 × 10^3^	0.876
Villitis of unknown etiology	1.07 × 10^4^	0.495	−6.91 × 10^2^	0.923	−262 × 10^4^	0.217

Reported models include all subjects, with cohort (CHD vs non-CHD) as a co-variate whose beta and p-value are reported above. The interaction term between each placental lesion and cohort was included in all models. Higher placental weight percentile was significantly associated with larger fetal intracranial volume. The presence of any pathology was associated with lower ICV with a significant interaction term indicating CHD moderates this effect. Any placental vascular malperfusion, either maternal, fetal, or both, was associated with significantly lower total fetal ICV. Fetal vascular malperfusion was also associated with lower total fetal ICV, however this result is interpreted with caution due to only four subjects in the non-CHD cohort with this placental lesion. *CHD* Congenital Heart Disease, *Non-CHD* No Congenital Heart Disease (typically developing). *Significant *p*-value < 0.05

**Fig. 2:**
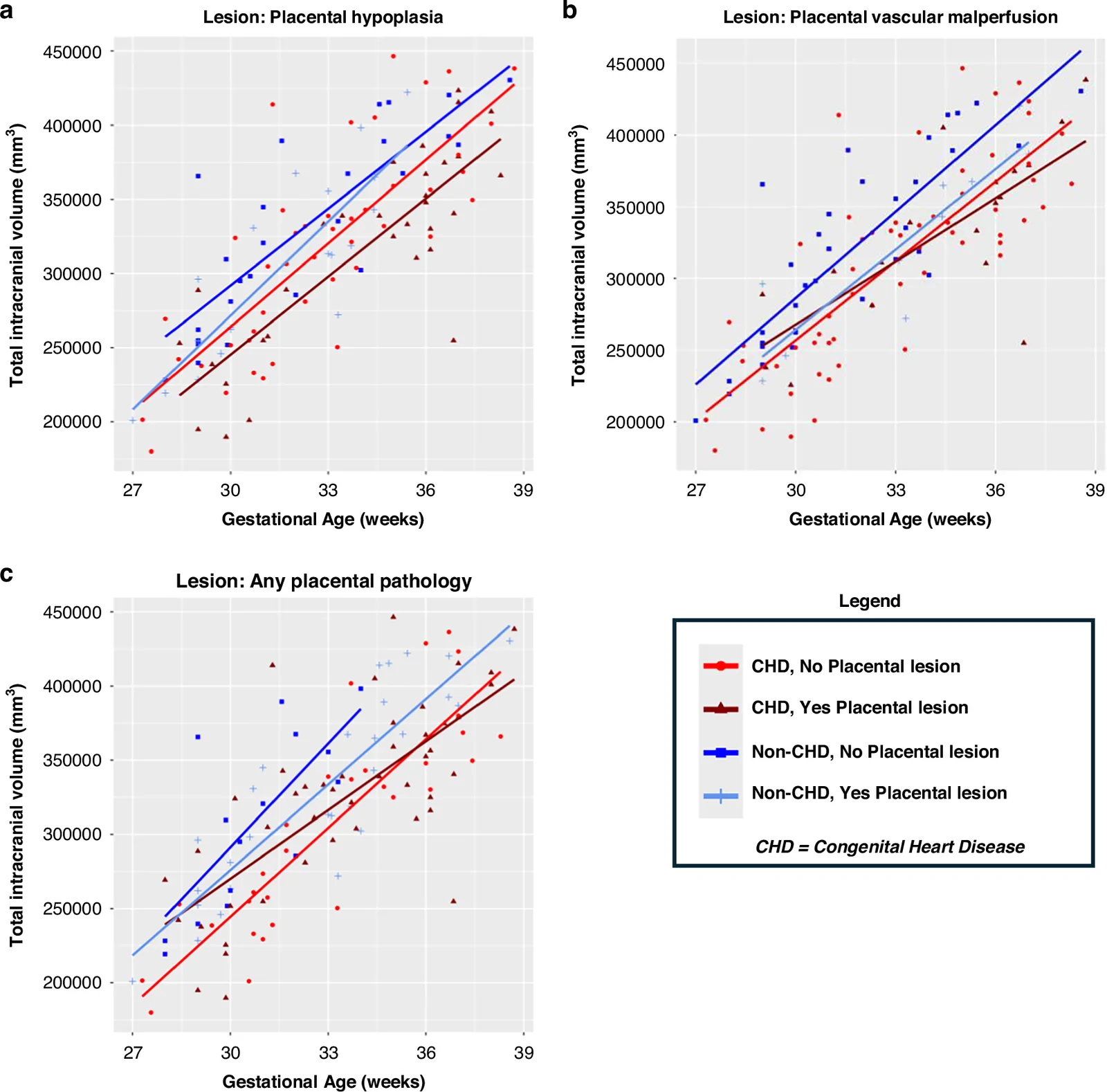
The effect of placental lesions on total fetal intracranial volume (ICV) in congenital heart disease (CHD) and non-CHD cohorts. **a** The presence of placental hypoplasia (defined as placental weight <10th percentile) was associated with significantly lower ICV in the CHD cohort (*p* = 0.007) and demonstrated a trend in the non-CHD cohort (p = 0.081). **b** Placental vascular malperfusion (either maternal, fetal, or both) was associated with lower ICV in stratified analysis of the non-CHD cohort (*p* = 0.012); the CHD cohort analysis was NS. **c** Presence of any placental pathology (one or more lesions present) was associated with decreased total ICV in the non-CHD cohort (*p* = 0.023). In the model examining the presence of any pathology and CHD to predict total ICV, inclusive of an interaction term for these two variables, there was a significant interaction between the presence of any pathology, and CHD (*p* = 0.023, also reported in Table 3).

Any placental lesion (placenta found to have one or more lesion), among non-CHD subjects, predicted a lower total ICV (β = –2.51 × 10^4^, 95% CI [−4.64 × 10^4^, −3.70 × 10^3^], *p* = 0.023) (Fig. 2). This finding was not seen in the CHD cohort.

Placental weight percentile was positively associated with total ICV in the combined cohort (β = 331.3, 95% CI [127.6, 534.0], *p* = 0.002). This association was also seen in the stratified analysis, (CHD cohort: β = 340.7, 95% CI [54.7, 626.6], *p* = 0.020 / non-CHD cohort: β = 333.9, 95% CI [35.1, 632.6], *p* = 0.030).

Placental hypoplasia was associated with lower total ICV in the combined cohort (β = −2.1 × 10^4^, 95% CI[−3.4 × 10^4^,−8.4 × 10^3^, *p* = 0.001], as well as upon stratified analysis (CHD cohort β = −2.4 × 10^4^, 95% CI[−4.1 × 10^4^, −6.7 × 10^3^], *p* = 0.007 / non-CHD cohort: β = −1.8×10^4^, 95% CI [−3.7 × 10^4^, 2.2 × 10^3^], NS: *p* = 0.081). The trajectory of fetal ICV by gestational age differs in the two cohorts (Fig. 2).

Any vascular malperfusion was associated with lower total ICV in the non-CHD cohort upon stratified analysis (β = −3.0×10^4^, 95% CI[−5.3 × 10^4^, −6.9 × 10^3^], *p* = 0.012) (Fig. 2).

### Sensitivity analysis to site variable: total fetal intracranial volume and placental pathology

In a sensitivity analysis of the main effect on ICV of placental pathology lesions, restricted to UPMC subjects, the four lesions demonstrating significant associations with fetal ICV were redemonstrated, and results are included in Supplementary Table 3.

Combined cohort and CHD only analyses re-demonstrate significance for all original analysis findings except for placental weight percentile, which was no longer significant in the CHD only cohort when only UPMC subjects were included:

Placental weight percentile was positively associated with total ICV in the combined cohort (β = 254.9, 95% CI [3.02, 506.8], *p* = 0.047). The CHD-only cohort is now NS when including only UPMC subjects, though the beta magnitude and direction were very similar to the full analysis (β = 214.7, *p* = 0.43).

Placental hypoplasia was associated with lower total ICV in the combined cohort (β = −1.9 × 10^4^, 95% CI[−3.4 × 10^4^,−3.3 × 10^3^, *p* = 0.018]), as well as upon stratified analysis (CHD cohort β = −3.2 × 10^4^, 95% CI[−5.8 × 10^4^, −5.7 × 10^3^], *p* = 0.020).

Any vascular malperfusion was associated with lower total ICV in the combined cohort (β = −2.8 × 10^4^, 95% CI[−4.7 × 10^4^, −8.7 × 10^3^], *p* = 0.005).

### Placental pathology and regional brain tissue volumes

Supplementary Table 2 lists regression model parameters for all significant associations between placental lesions and regional brain volumes with inclusion of an interaction term between cohort and placental lesion in predicting the regional brain volume. Combined cohort (both CHD and non-CHDs) and analyses stratified by cohort are presented below:

Placenta weight percentile was inversely associated with normalized hippocampus amygdala complex in the non-CHD cohort (β = −7.74 × 10^−^^6^, 95% CI [−1.44 × 10^−^^5^, −1.06 × 10^−^^6^], *p* = 0.024) as well as normalized brain stem volume in the combined cohort (β = −1.36 × 10^−^^5^, 95% CI [−2.41 × 10^−^^5^, −3.17 × 10^−^^6^], *p* = 0.011) and non-CHD cohort (β = −1.82 × 10^−^^5^, 95% CI [−3.46 × 10^−^^5^, −1.72 × 10^−^^6^], *p* = 0.031).

Any vascular malperfusion was associated with larger normalized deep grey matter (β = 3.7 × 10^−^^3^, 95% CI [1.33 × 10^−^^4^, 7.42 × 10^−^^3^], *p* = 0.043) in the non-CHD cohort. Extra-axial cerebrospinal fluid volumes were lower in the combined cohort (β = −2.17 × 10^−^^2^, 95% CI [−3.72 × 10^−^^2^, −6.24 × 10^−^^3^], *p* = 0.006), CHD subjects (β = −2.76 × 10^−^^2^, 95% CI [−5.12 × 10^−^^2^, −4.01 × 10^−^^3^], *p* = 0.022), and non-CHD subjects with any form of vascular malperfusion (β = −1.30 × 10^−^^2^, 95% CI [−2.32 × 10^−^^2^, -2.89 × 10^−^^3^], *p* = 0.013).

Maternal vascular malperfusion was associated with lower extra axial cerebrospinal fluid volume in the CHD (β = −3.64 × 10^−^^2^, 95% CI [−7.08 × 10^−^^2^, −1.09 × 10^−^^3^], *p* = 0.039), non-CHD (β = −1.20 × 10^−^^2^, 95% CI [−2.37 × 10^−^^2^, −3.24 × 10^−^^4^], *p* = 0.044), and combined cohorts (β = −2.31 × 10^−^^2^, 95% CI [−4.34 × 10^−^^2^, −2.69 × 10^−^^3^], *p* = 0.027). Normalized cortical plate volume was higher in the CHD cohort in the presence of maternal vascular malperfusion (β = 2.06 × 10^−^^2^, 95% CI [3.78 × 10^−^^3^, 3.73 × 10^−^^2^], *p* = 0.017).

Chorangiosis was associated with smaller normalized cortical plate volumes (β = −8.84 × 10^−^^3^, 95% CI [−1.67 × 10^−^^2^, 1.01 × 10^−^^3^], *p* = 0.027) in the combined cohort, as well as trending smaller in the CHD cohort (β = −1.08 × 10^−^^2^, *p* = 0.068). It was also positively associated with normalized extra-axial cerebrospinal fluid volumes in the combined cohort (β = 1.79 × 10^−^^2^, 95% CI [2.83 × 10^−^^3^, 3.29 × 10^−^^2^], *p* = 0.020).

Analysis by CHD subtype of 1- versus 2-ventricle physiology was performed to compare the effects of placental pathologies on total ICV by ventricle subtype. No significant differences were observed between these subtypes.

### Placental pathology relationships

**Fig. 3:**
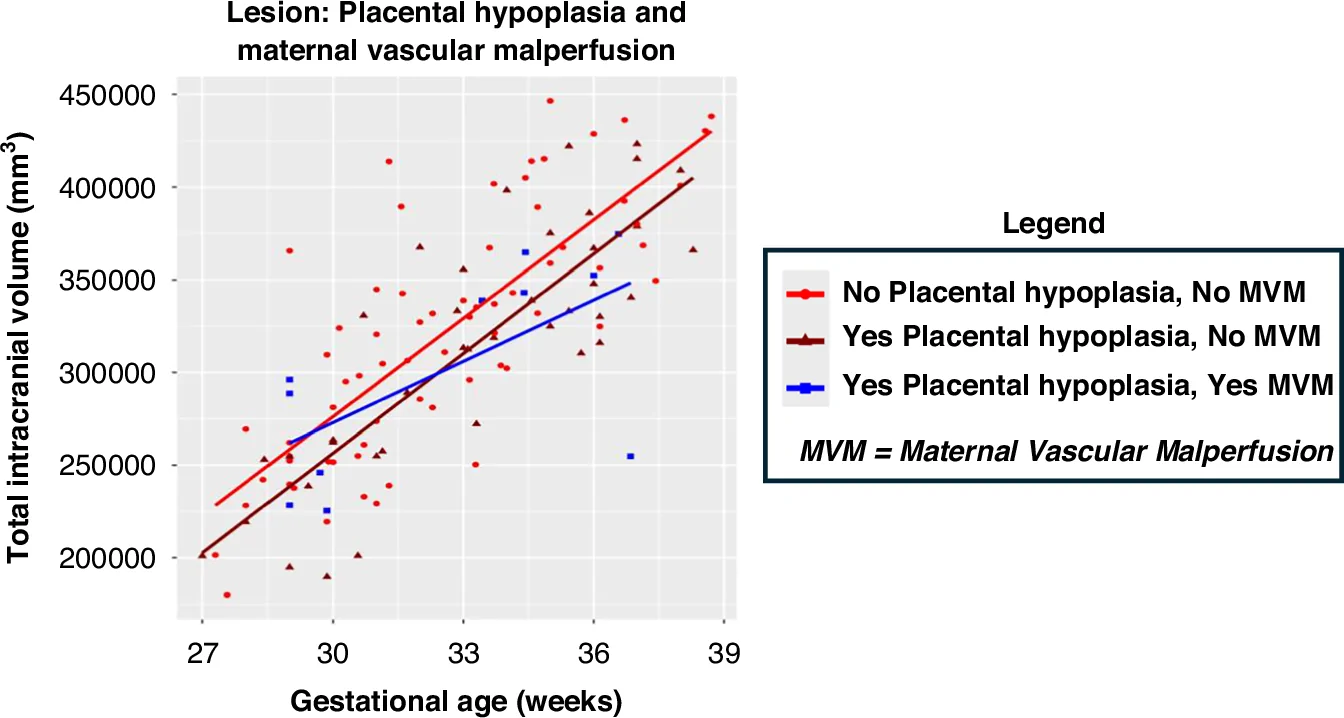
The effect of placental hypoplasia and maternal vascular malperfusion lesions on total fetal intracranial volume (ICV) is shown here. Placental hypoplasia is part of the constellation of features used to diagnose maternal vascular malperfusion via the Amsterdam criteria. Using linear regression analysis, the two lesions’ contributions to total ICV were examined. We found that compared to participants with neither lesion (*N* = 68), those having placental hypoplasia *with* maternal vascular malperfusion had the smallest ICVs (*N* = 11, β = −2.56 *p* = 0.024). The presence of placental hypoplasia *without* maternal vascular malperfusion was also significant (*N* = 39, β = −2.08 × 10^4^, *p* = 0.003), though the larger absolute beta value when both lesions are present suggests additive mal effects to fetal brain development from maternal vascular malperfusion on a poorly grown placenta. Analyses for participants with maternal vascular malperfusion without placental hypoplasia were non-significant, likely due to low N (N = 3).

The presence of maternal vascular malperfusion was independently associated with lower placental weight percentile (present: 13.3 ± 31.2 vs absent: 32.2 ± 30.9, *p* = 0.030). Post-hoc examination of total ICV in participants with neither, ie no maternal vascular malperfusion and no placental hypoplasia (N = 68, referent), versus those with placental hypoplasia *and* maternal vascular malperfusion (*N* = 11, β = −2.56 × 10^4^, 95% CI [−4.78 × 10^4^, −3.43 × 10^3^], *p* = 0.024), and versus those with placental hypoplasia *without* maternal vascular malperfusion (*N* = 39, β = −2.08 × 10^4^, 95% CI [−3.45 × 10^4^, −6.99 × 10^3^], *p* = 0.003) were both significantly lower, and a larger change in ICV seen when both lesions were present (Fig. 3). Comparison of participants with maternal vascular malperfusion without placental hypoplasia (*N* = 3) was non-significant. Examination of this variable against regional brain volumes was also non-significant.

## Discussion

Our study is the first to report the association of individual histopathological placental lesions, utilizing the Amsterdam criteria, on fetal brain volumes. It is known that decreased total fetal brain volume is associated with poor neurodevelopmental outcomes in CHD.^25^ Our findings suggest that some pathologies are worse for the fetal brain than others, a novel insight into the role of placental dysfunction on the ontogeny of adverse neurodevelopment in CHD.

### Congenital Heart Disease and Placental Pathology

Prior studies have found a higher incidence of placental pathologies in subjects with CHD versus those without,^16,18,28^, particularly malperfusion lesions, delayed villous maturation, and chorangiosis. We did not observe statistically significant differences in placental pathology incidence between CHD and non-CHD cohorts. This may be due to limited sample size, as well as the non-CHD group’s high rate of hypertensive disorders, which are associated with placental hypoplasia and vascular malperfusion lesions^40–43^ We did observe a trend toward increased incidence of fetal vascular malperfusion (8.9% versus 14%) as well as its hallmark histopathological feature, avascular villi (11% versus 21%) in the CHD cohort. The association of these placental findings with CHD has been reported elsewhere.^18^

### Congenital heart disease and fetal brain development

We found that placental vascular malperfusion and placental weight are associated with decreased total fetal ICV. These lesions implicate changes in blood flow as a primary driver of the link to reduced fetal brain volume.^23^ Maternal vascular malperfusion,^44^ fetal vascular malperfusion,^45^, and placental hypoplasia^46^ have all been associated with reduced blood flow to the developing fetus. Placental hypoplasia’s association with chronic fetal hypoxia and poor growth is well described.^47^ In the CHD population, abnormal placental perfusion has been found via MRI when compared to non-CHD.^24^ Sun et al found that umbilical vein oxygen content is lower in the CHD placenta, indicating the organ delivers hypoxic blood to the fetus.^48^ In addition to impacting oxygen delivery, flow reduction affects substrate delivery. Cromb et al recently reported that cardiac lesions expected to most severely affect substrate delivery to the fetal brain resulted in smaller total and regional volumes.^49^

Though not measured in our study, it is likely that decreased substrate delivery and tissue oxygen delivery (DO2) from placenta to fetus are part of the pathophysiologic mechanism behind reduced brain volumes, particularly in CHD. Further studies measuring in vivo placental and fetal oxygen delivery, especially to the fetal brain, would add insight into this potential mechanism.

In the CHD cohort we observed a drop off in total fetal intracranial volumes in early third trimester (28-32 weeks period), which aligns with prior work.^48,50^ Placental redundancy relative to fetal size may preclude mal effects until the fetal brain experiences rapid growth in the third trimester.^51^ The metabolic demand during this period may outpace placental support in the presence of vascular compromise. We found that the dual effect of CHD plus a placental lesion resulted in the smallest total ICV. In a recent study, placental pathology severity (via a severity scoring system) was correlated with reduced early post-natal total brain, cerebellum, and cortex volumes in CHD subjects.^20^ Consistent with this, we found that any placental pathology versus none, was associated with smaller total fetal ICV, moderated by presence of congenital heart disease. This suggests that placental abnormalities in general are associated with detectable alterations in fetal brain growth.

The significant interaction term between any placental pathology and CHD may reflect diminished capacity in the CHD population for appropriate fetal compensation in the setting of in-utero adversity from placental hypoplasia and/or malperfusion. Doppler studies of cerebral blood flow in CHD have demonstrated vasodilation of cerebral vasculature, but a compensatory effect that is insufficient to improve cerebral oxygen consumption.^23^ We note in our results that in the setting of placental hypoplasia, non-CHD subjects appeared to have total ICV approaching peers as gestation progressed, suggesting physiologic compensation, whereas in CHD subjects this was not observed (Fig. 2, panel a). This is consistent with previous findings that even in the presence of placental hypoplasia head and body weight percentiles are concordant, suggesting against brain sparing physiology in fetuses with CHD.^52^ Placental hypoplasia is often a feature of maternal vascular malperfusion, hence the examination of these two lesions in more depth. We found that in the presence of placental hypoplasia, maternal vascular malperfusion had an increased effect on the fetal brain (more negative beta), suggesting additive effects from these two lesions. Though biologically plausible and clinically relevant, placental hypoplasia and vascular malperfusion may be related to upstream pathologies such as fetal/maternal genotype and unaccounted for pre-pregnancy maternal co-morbidities present prior to or early on post-conception. We acknowledge that the impact of these lesions on CHD fetuses cannot distinguish between solely placental vs other shared upstream effects.

The relationship between lower brain volumes and poorer neurodevelopmental outcomes in the CHD population has been demonstrated in multiple studies using post-natal MRI.^53–55^ Furthermore, there is a positive correlation between smaller fetal and neonatal brain volumes in CHD and associated neonatal brain injury.^56^ Improved in-utero brain growth may help reduce pre- and post-natal brain injury in the vulnerable CHD population, where the neonatal brain is exposed to insults such as major surgery and cardiopulmonary bypass.

### Placental pathology and regional brain volumes

A general pattern emerges in our results in which a placental pathology is associated with an increased regional brain volumes to ICV ratio. We found this for hippocampus amygdala complex, brainstem, and deep grey matter. Deep cerebral structures have been shown to have a less rapid growth rate and overall size fold change during development than other brain regions, such as white matter and cortex.^51^ It may be that lower ICV in these subjects is driving the differences seen in regional brain volumes. These results are exploratory and hypothesis-generating, however definitive determination about whether these structures are falling off in terms of growth will require a longitudinal study utilizing a minimum of two volumetric data points per subject to compare growth rates. At least one post-natal imaging point would add further clinical relevance as slowed head growth trajectory in early childhood is associated with poor neurodevelopment,^57–61^ a process that likely starts in-utero.

### Strengths and limitations

Our study has several strengths. Placental reports were blinded to study group, and importantly, the Amsterdam criteria were followed closely for rigor and reproducibility of results. With respect to imaging analyses between two sites, both used harmonized protocols and analysis pipelines to minimize site-specific differences, and the site was accounted for in all statistical models run. This study provides earlier brain imaging, from mid- to late gestation, compared to existing literature on this subject. Important known confounders of fetal brain volumes and placental lesions were examined for each model, and a lower end of threshold for confounding (10%) was used. Prospective study design, as well as inclusion of non-CHD subjects, allowed us to make direct comparisons with respect to the presence of CHD and the presence of specific placental lesions. Overall, our CHD and non-CHD cohorts were well matched on important neonatal variables, including placental weight, gestation age at birth, and birthweight. They were also well matched on rates of primary placental anomalies reported upon, i.e., vascular malperfusion and placental hypoplasia, which allowed us to examine differences in the impact of these lesions on brain volumes between fetuses with CHD and those without.

This study also had limitations. In stratified analyses in which CHD and non-CHD cohorts were analyzed separately, a small sample size may have led to being underpowered. We were also underpowered to analyze CHD lesion subtypes beyond 1 versus 2 ventricle physiology. The non-CHD cohort had a higher rate of maternal hypertensive disorders compared to the CHD cohort. This is likely related to obstetric indications needed for sending non-CHD placentas for pathology. This may have led to a type II error, particularly with respect to the placental variables associated with hypertensive disorders of pregnancy, maternal vascular malperfusion, and placental hypoplasia. All lesions in this study were categorized by a placental pathologist (PZ) using clinical pathology reports. However, reports can be subjective and may vary between pathologists and institutions regarding the diagnosis, grading, and staging of injury patterns.

## Conclusion

Placental hypoplasia and vascular malperfusion are associated with decreased total intracranial volume in utero. Fetuses impacted by CHD with concurrent placental lesions are particularly susceptible and demonstrate the smallest total intracranial volumes. Our study is unique because we utilized standardized placental pathology diagnoses and a prospective study design inclusive of a non-CHD group. Future directions include functional in-utero vascular imaging of the placenta to develop biomarkers for poor placental function that correlate with post-natal histopathology and fetal and post-natal outcomes.

Our work links placental health to fetal brain development, adding to the growing body of evidence implicating the in-utero environment in poor neurodevelopment in CHD. Addressing placental function may be an avenue toward improving neurodevelopmental outcomes.

## Supplementary information

Supplementary Information

## Data Availability

The derived data presented in this study are available upon reasonable request from the corresponding author. Raw data are not publicly available due to patient privacy.
