# Supplementary material for: Placental hypoplasia and vascular malperfusion are associated with reduced brain volumes in fetal congenital heart disease
Source: Pediatr Res. 2026 Jan 7;100(3):1266–75. doi: 10.1038/s41390-025-04698-3 (PMC13672080; doi:10.1038/s41390-025-04698-3)
Supplement: Supplementary file 1 [file 41390_2025_4698_MOESM1_ESM.pdf]

Supplemental Table 1: Congenital Heart Disease Cohort Lesion Types

	**Total N=76**<br>**N (%)**
**2-Ventricle Cardiac Defects**	
Aortic Stenosis	1 (1.3%)
AVSD + aortic arch hypoplasia	1 (1.3%)
Complex heterotaxy, AVSD	2 (2.6%)
D-TGA and variants	13 (17.1%)
Isolated aortic arch anomaly	5 (6.6%)
L-TGA (with VSD, coarctation or PA)	3 (3.9%)
Systemic venous anomaly	1 (1.3%)
Tetralogy of Fallot	8 (10.5%)
Total anomalous pulmonary venous return	1 (1.3%)
Truncus arteriosus	1 (1.3%)
**1-Ventricle Cardiac Defects**	
Ebstein’s anomaly	3 (3.9%)
Double inlet left ventricle + arch hypoplasia	2 (2.6%)
Hypoplastic left heart syndrome	18 (23.7%)
Hypoplastic right heart syndrome	1 (1.3%)
Single ventricle with PS/PA	9 (11.8%)
Other single ventricle	7 (9.2%)

AVSD = atrioventricular septal defect; CHD = congenital heart disease; D-TGA = Dextro Transposition of the Great Arteries; L-TGA = Levo Transposition of the Great Arteries, PA = pulmonary atresia, PS = pulmonary stenosis, VSD = ventricular septal defect

Supplemental Table 2. Effect of Placental Lesions on Regional Brain Volumes

Placental Lesion	Brain Region	Main Effect on Regional Brain Volume		Cohort = CHD (vs non-CHD)		Interaction	
		Beta	p	Beta	p	Beta	p
Placental Efficiency	Cerebellum	-1.10×10^{-3}	**0.047***	-3.81×10^{-3}	0.398	1.18×10^{-3}	0.061
Any Vascular Malperfusion	Deep Grey Matter	3.85×10^{-3}	**0.028***	9.38×10^{-4}	0.364	-6.02×10^{-3}	**0.006***
Any Pathology	Brainstem	1.20×10^{-3}	**0.039***	2.18×10^{-3}	**<0.001***	-1.33×10^{-3}	0.064
Chorangiosis	Intra-ventricular fluid	-2.01×10^{-3}	0.151	-1.70×10^{-3}	0.064	3.82×10^{-3}	**0.034***
Fetal Vascular Malperfusion	Deep Grey Matter	7.63×10^{-3}	**0.003***	6.40×10^{-4}	0.496	-1.04×10^{-2}	**<0.001***
Fetal Vascular Malperfusion	Hippocampus Amygdala Complex	1.01×10^{-3}	**0.030***	2.85×10^{-4}	0.101	-9.95×10^{-4}	0.064

Supplemental Table 2. Significant associations between normalized regional brain volumes and placental lesions with interaction term in the model are shown here. Regional brain volumes were normalized to total intracranial volume as a ratio (i.e. regional brain volume/total intracranial volume). Reported models include all subjects, with cohort (CHD or non-CHD) as a co-variate. Values in the cohort column reflect the influence of CHD; the non-CHD group was referent. The interaction term was examined between each placental lesion and cohort (CHD vs non-CHD) status. Placental variables examined were: Maternal vascular malperfusion, fetal vascular malperfusion, villitis of unknown etiology, avascular villi, chorangiosis, and placental weight percentile, as well as four created variables: 1) "placental hypoplasia", defined as a placental weight <10th %ile, 2) "any vascular malperfusion," defined as maternal and/or fetal vascular malperfusion, 3) "any pathology," defined as presence of one or more placental lesions, and "placental efficiency" which is the value of birth weight (g) divided by placental weight (g). The regional brain volumes examined were cortical plate, white matter, hippocampus amygdala complex, deep grey matter, cerebellum, brainstem, extra-axial cerebrospinal fluid, and intraventricular cerebrospinal fluid. Only results with significant associations are listed, for brevity. * Significant p-value <0.05

Supplemental Table 3. Sensitivity Analysis of Site Variable on Main Effect Findings

Placental Lesion	Main Effect on Total Intracranial Volume		Cohort = CHD (vs non-CHD)		Interaction	
	Beta	p	Beta	p	Beta	p
**Placental weight percentile**	3.22×10^2	**0.040***	-2.03×10^3	0.873	-1.26×10^2	0.6813
Placental hypoplasia	-1.45×10^4	0.142	2.55×10^3	0.826	-2.00×10^4	0.265
Placental efficiency	2.67×10^3	0.568	-3.42×10^4	0.570	3.66×10^3	0.659
**Any pathology present**	-2.27×10^4	**0.041***	-2.43×10^4	0.127	2.48×10^4	0.196
Acute chorioamnionitis	8.98×10^3	0.425	-1.79×10^3	0.235	-1.90×10^4	0.362
**Any vascular malperfusion**	-2.69×10^4	**0.028***	-1.18×10^4	0.235	1.48×10^4	0.539
Maternal Vascular Malperfusion	-1.38×10^4	0.329	-1.08×10^4	0.266	4.16×10^4	0.278
**Fetal vascular malperfusion**	-4.31×10^4	**0.016***	-8.65×10^3	0.349	1.08×10^4	0.715
Chorangiosis	-1.41×10^4	0.252	-1.43×10^4	0.223	2.12×10^4	0.309
Avascular villi	-5.42×10^3	0.733	-2.46×10^3	0.799	-3.14×10^4	0.234
Villitis of unknown etiology	1.11×10^4	0.458	-3.41×10^3	0.728	-2.57×10^4	0.326

Supplemental Table 3. Sensitivity analysis of the site variable upon main effect of placental associations with total intracranial volume (ICV). Only one site, University of Pittsburgh Medical Center (UPMC), is included in the above analyses (the multi-site analysis inclusive of CHLA subjects is shown in **Table 3**). Associations between total ICV and placental lesions with interaction term in the model are shown here. Values in the cohort column reflect the influence of CHD; the non-CHD group was referent. As in the main analysis, the same four placental lesions demonstrate a significant association with total ICV: Placental weight percentile, any pathology present (inclusive of any placenta for which one or more lesion is found), any vascular malperfusion (maternal, fetal, or both), and fetal vascular malperfusion. The magnitude and direction of the beta values for all four lesions are maintained in this sensitivity analysis. The consistency of effect estimates indicates that placental impacts on ICV are not attributable to differences in site. CHD = Congenital Heart Disease, Non-CHD = No Congenital Heart Disease (typically developing). *Significant p-value <0.05.
